# An Effective COVID-19 Medical Student Elective

**DOI:** 10.5811/westjem.2021.11.53656

**Published:** 2022-01-03

**Authors:** Gabriel Sudario, Warren Wiechmann, Julie Youm, Khanh-Van Le-Bucklin

**Affiliations:** * University of California, Irvine, Department of Emergency Medicine, Irvine, California; † University of California, Irvine, School of Medicine, Medical Education, Irvine, California

## Abstract

**Background:**

The COVID-19 pandemic has revealed the importance of teaching medical students pandemic preparedness and COVID-19 related clinical knowledge. To fill the gap of COVID-19 instruction backed by evaluation data, we present a comprehensive COVID-19 pilot curriculum with multiple levels of evaluation data.

**Methods:**

In the spring of 2020, the University of California, Irvine (UCI) School of Medicine piloted a two-week, primarily asynchronous COVID-19 elective course for medical students. The goal of the course is to provide a foundation in clinical care for COVID-19 while introducing students to emerging issues of a modern pandemic. Objectives align with institutional objectives, and instruction is delivered in thematic modules. Our curriculum utilizes numerous instructional strategies effective in distance learning including independent learning modules (ILM), reading, video lectures, discussion board debates, simulation and evidence-based argument writing. We designed a three-level, blended evaluation plan grounded in the Kirkpatrick and Kirkpatrick evaluation model that assessed student satisfaction, relevance, confidence, knowledge and behavior.

**Results:**

Our end of course survey revealed that students had high levels of satisfaction with the curriculum, and felt the course was relevant to their clinical education. Various assessment tools showed excellent levels of knowledge attainment. All respondents rated themselves as highly confident with the use of personal protective equipment, though fewer were confident with ventilator management.

**Conclusion:**

Overall our pilot showed that we were able to deliver relevant, satisfying COVID-19 instruction while allowing students to demonstrate knowledge and desired behaviors in COVID-19 patient care.

## BACKGROUND

The COVID-19 pandemic has created unprecedented changes to healthcare and medical education systems. As case numbers in the United States rose exponentially in March 2020, many medical schools developed COVID-based electives to give students relevant clinical experiences while responding to the Association of American Medical Colleges recommendation to “[pause] all student clinical rotations.”^1^ Despite this pause being lifted, the current pandemic reveals the importance of teaching medical students pandemic preparedness along with the tenets of COVID-19 patient care. A recent meta-analysis by Ashcroft et al. showed that implementing disaster training into undergraduate medical education improved student knowledge, skills, and preparedness during times of a pandemic.^2^ Therefore, pandemic preparedness education provides training that is relevant through the current pandemic, as well as for future practice.

While some medical schools developed COVID-19 related courses, a thorough literature review found no medical student course that has undergone a formal evaluation process. To bridge this unmet need, we present a comprehensive, multidisciplinary, COVID-19, medical student pilot curriculum with multiple levels of evaluation data. To our knowledge, this novel course is the first to combine formats of virtual learning, simulation, independent learning modules, moderated discussions, and service learning to meet course goals and objectives.

In the spring of 2020, the University of California, Irvine (UCI) School of Medicine developed and implemented a primarily asynchronous, two-week COVID-19 elective course for medical students. The course was designed within the Kern framework of curricular development.^3^ Based on a needs assessment consisting of focus groups with course leadership, medical school curricular affairs leadership (associate dean for clinical science and vice dean of medical education) and medical students at various levels, we developed the instructional goal to provide a foundation in clinical care for COVID-19 patients while introducing students to emerging issues of a modern pandemic including ethical dilemmas, palliative care, tele-health, personal mental/physical health strategies, and community service.

## OBJECTIVES

Using frameworks from Dick, Carey and Carey in *The Systemic Design of Instruction*, we created thematic modules matching the instructional goals of our needs assessment, wrote terminal objectives for each thematic module, and mapped these objectives to the School of Medicine’s program objectives and competencies (see Appendix 1).^4^ Within each module, we also created subordinate objectives to guide the selection of instructional strategies and materials (Table 1).

## CURRICULAR DESIGN

### Educational Strategies

The course employs a wide range of educational strategies that align with the various domains of Bloom’s taxonomy.^5^ These include independent reading, videos, podcasts, team-based learning, discussion board forums, standardized patient encounters, debate, and simulation. Instructional material was curated by analyzing and validating existing content developed by outside educational institutions. This included complete review from the UCI School of Medicine Curriculum and Educational Policy Committee. A full accounting of instructional strategies and materials aligned with learning objectives can be seen in Table 1.

### Implementation

A pilot course was run in April 2020 with 51 medical students at the School of Medicine through the Canvas learning management system (Instructure, Inc., Salt Lake City, UT). Of the 51 medical students, 67% (n = 34) had just completed their second year of medical school; 24% (n = 12) had just completed their third year, and 10% (n = 5) of students were in the final month of their fourth year of medical school. The course was held in a hybrid format with most sessions held virtually and/or asynchronously. Students did attend an in-person, socially distanced, simulation and personal protective equipment sessions during the course.

### Assessment and Evaluation Tools

We used the Kirkpatrick and Kirkpatrick model of evaluation, with a focus on Levels 1–3, to design our various measurement instruments.^6^ Due to the pause on clinical rotations, Level 4 evaluation of clinical outcomes was not pursued. This study qualified as exempt by the UCI Institutional Review Board.

### Level 1: Reaction

At the conclusion of the elective, students completed a course evaluation that was administered through the Qualtrics survey platform (Qualtrics, Provo, UT) and distributed via email. The evaluation tool contained 27 questions asking students to rate the course (on a four-point Likert scale) in domains of satisfaction, teaching quality, objectives, instructional materials, confidence, and relevance of the instructional content. The complete course evaluation is available in Appendix 2.

### Level 2: Knowledge

To assess knowledge, students were assessed with four multiple-choice quizzes ranging from 10–20 questions in length. These quizzes were developed using assessment items that align with content objectives and were piloted with this learning group. We used two additional assignments to assess Level 2 outcomes and higher orders of thought:

- Epidemiology Visualization Assignment: Students created a novel graphic/visualization of COVID-19 epidemiologic data. A five-point grading rubric was used to assess the student’s ability to apply epidemiology principles to real-life data.
- Policy Position Statement: Students created a short position statement for the reopening of schools in their local county. A five-point rubric was created to assess their ability to integrate evidence-based principles into an effective written argument.

### Level 3: Behavior

We assessed behavioral outcome data through various assignments, discussions, and simulation sessions throughout the course:

- Appraisal of Emerging Literature Assignment: Students were asked to create an infographic or written report of the evidence backing a certain side of a controversial debate related to COVID-19 care. A five-point grading rubric was created to assess student work in the domains of medical evidence, effective written/visual communication, and professionalism.
- Simulation Session: Students participated in a simulation session that focused on the care of a critically ill COVID-19 patient. Simulation instructors used a 15-point, critical action checklist to assess student competence in patient care. The simulation case and critical actions checklist were implemented from a peer-reviewed, published case scenario.^7^
- Standardized Patient Encounter: Students participated in two virtual standardized patient encounters. Students were evaluated with an eight-point critical action checklist.
- Discussion Board Participation: Five discussion board activities were required of students participating in our elective. A participation rubric was created to ensure timely, meaningful conversation between students. Discussion boards were graded by the course director throughout the two-week course.

All assignments and grading rubrics are available in Appendix 3.

## IMPACT/EFFECTIVENESS

### Results

We collated all evaluation data and analyzed student scoring through simple percentage comparisons of various scoring categories. All student scores for various assignments were compiled, and the mean and standard deviation of scores were calculated to analyze the data. We categorized results based on the Kirkpatrick and Kirkpatrick hierarchy.^6^

### Level 1 Results

**Figure 1:**
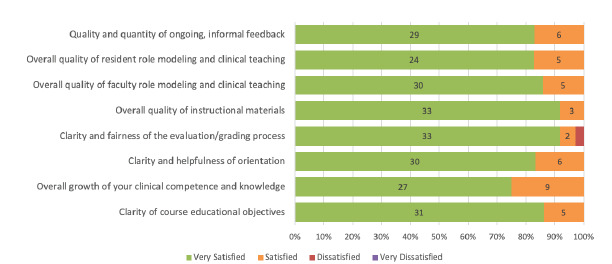
Post-course survey responses related to student satisfaction in various instructional categories (n=36).

Seventy-one percent (n = 36) of students completed the post-course evaluation. Virtually all respondents in the post-course evaluation rated their satisfaction in various aspects of the course as “satisfied” or “very satisfied” (Figure 1). Notably, 92% (n = 33) of students rated their satisfaction with the quality of instructional materials as “very satisfied;” and 100% (n = 36) of students rated their satisfaction with the overall growth of clinical competence and knowledge as “very satisfied” or “satisfied.”

When asked to rate agreement with the statement “this course has prepared me to better understand the complex information related to emerging pandemics,” 86% of students (n = 31) “strongly agreed” with the statement while 14% (n = 5) of students “agreed” with the statement. Similar levels of agreement were shown with students’ ability to implement tools from this course into future work, and the value of this course as a clinician overall.

### Level 2 Results

#### Knowledge

The mean score of knowledge quizzes was 90.5% (n = 51, standard deviation [SD] 0.89) showing excellent knowledge attainment. Students scored equally well in topics of clinical care, epidemiology, radiology, and PPE use.

#### Confidence

**Figure 2:**
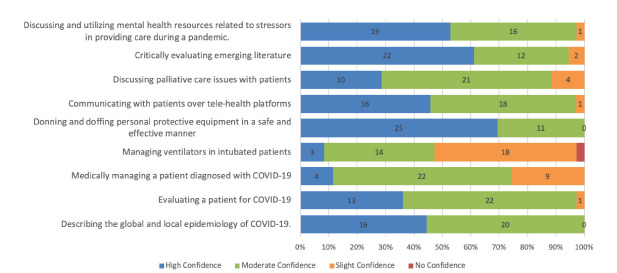
Post-course survey responses related to student confidence (n=36).

Our survey evaluated confidence in various domains of pandemic patient care. When surveyed about confidence in donning and doffing personal protective equipment, 100% (n = 36) of students rated “high” or “moderate” confidence in this task. Students also exhibited similar confidence in describing the epidemiology of COVID-19, critically evaluating literature, and discussing mental health issues/resources (Figure 2). In contrast, only 47% (n = 17) of students rated “high” or “moderate” confidence in ventilator management, and only 72% (n = 26) students rated “high” or “moderate” confidence in medical management of COVID-19 patients.

### Level 3 Results

#### Literature Appraisal

All students submitted the literature appraisal assignment on time. Of the five points in the assignment, the average score was 4.99 (n = 51, SD 0.07).

#### Simulation Session

All 51 students participated in the COVID-19 patient simulation session. Due to various constraints, students participated in the session in groups of five. Because the simulation facilitator could not individually evaluate students, the critical actions checklist was applied at the group level. All groups either completed all critical actions or took time to debrief on the missed critical actions in the checklist.

#### Standardized Patient Encounters

In groups of three to four, all students (n = 51) had the opportunity to interview standardized patients in mock telehealth Zoom encounters (Zoom Video Communications, San Jose, CA). Small-group facilitators applied the critical action checklist to the group interview and reported that groups either completed all critical actions, or missed items were debriefed after interviews.

#### Discussion Board Participation

All 51 students actively participated in various discussion board topics. To receive full credit, students were required to contribute meaningful comments to each discussion. The average score for all discussion board assignments was 4.97 (n = 51, SD 0.04). Based on review of these discussions, students were able to demonstrate the following behavior:

- Appraise ethical dilemmas in COVID-19 patient care
- Prepare scripts for discussing end-of-life care with patients
- Appraise emerging literature related to COVID-19
- Share various coping tools related to mental well-being of COVID-19 providers
- Serve Orange County, CA, through community service opportunities.

#### Successes

Throughout the COVID-19 pandemic, medical students have expressed the desire “to be prepared to provide care,” while schools have struggled with “finding the best way to educate in the current climate.”^8^ Our course provides a highly rated framework for addressing this vital need. Our results show that students were able to gain key knowledge in COVID-19 patient care, exhibit behaviors necessary to work effectively in the time of a pandemic, and were overall satisfied with the learning experience. To our knowledge, our course is the first to show positive outcomes in knowledge acquisition, student confidence, and behavior.

By designing an experience with a breadth of educational strategies, we were able to ensure continued engagement throughout our elective, which was delivered almost exclusively over remote platforms. Course discussion boards required social negotiation and collaboration to discuss complex problems. Because students were processing and synthesizing emerging COVID-19 data in real time with the medical community, our course leveraged educational strategies grounded in constructivist learning theory, which states that “knowledge is constructed by learners as they attempt to make sense of experiences.”^9^

This course provides a generalizable framework for the delivery of future COVID-19 related and pandemic preparedness curricula. While the various curriculum objectives can be easily modified to introduce emerging topics such as social media disinformation and vaccine hesitancy, our hybrid delivery method and education strategies have proven to be successful in helping learners from various levels explore topics in pandemic preparedness and care. This curriculum is also generalizable to future outbreaks of infectious disease on the local or international level. While this curriculum focuses on COVID-19 patient care, topics in epidemiology, telehealth delivery, ethics, palliative care, PPE, and appraisal of emerging literature are easily applicable to other pandemic preparedness situations.

#### Challenges

Our evaluation did reveal some limitations in our curriculum. As described in our outcomes, students did rate lower levels of confidence in COVID-19 medical and ventilator management. This could be explained by the lack of clinical experience of most students in the class. The majority of students in the class were newly risen third-year medical students who had limited experience clinically in the hospital. General medical and respiratory management are skills learned after years on the medical ward.

Our pilot course also revealed weaknesses in some of our assessment tools. The average score of our literature and discussion board assignments were very high in our pilot cohort. While this can suggest very high engagement in our course activities, it also reveals that our assessment tools may have less validity in assessing academic attainment in the domain areas they were addressing.

#### Next Steps

The UCI School of Medicine COVID-19 medical student elective was an effective and satisfying asynchronous experience for medical students at our institution. Next steps in full implementation include further integration of immersive experiences in COVID-19 patient care, including augmented and virtual reality, task trainers and hands-on work with ventilators. We also plan to evaluate our assessment tools in various reliability and validity measures to better assess knowledge and behavior in our cohorts.

## Supplementary Information







## Figures and Tables

**Table 1:** Detailed learning module themes, module terminal objectives, subordinate objectives, instructional strategies, and activities.

Module theme and terminal objective	Subordinate objectives	Instructional strategies	Activity and materials
**Clinical care** The student will be able to recognize the clinical presentation of a COVID-19 patient, select and interpret diagnostic tests, and explain interventions for effective treatment of COVID-19 patients.	- Describe incubation period. - Describe typical clinical course. - Recognize risk factors for severe disease. - List imaging modalities used in the COVID-19 workup. - Describe lab abnormalities associated with disease. - Describe lab abnormalities related to increased mortality. - Synthesize the practical approach to patient management.	Independent learning module (ILM) Video lectures Simulation	Harvard Medical School (HMS) Course- Module 1 Osmosis- COVID-19 Disease video Lecturio COVID Overview videos COVID-19 simulated patient encounter
**Virology and epidemiology** The student will be able to explain general and specific mechanisms by which the SARS-CoV-2 virus causes disease and be able to describe its epidemiology.	- Identify the virus class. - Describe mechanism of infection. - Explain significance of Ro in viral infections. - Compare/contrast COVID-19 infection to annual influenza infection. - Define the epidemiology variables E, p and Nd. - Label a case growth curve with the following terms: inflection point, growth factor. - Describe why COVID-19 infections follow a logistic curve. - Create a logistic curve for a given country’s infection data. - Create a visualization of Orange County, CA, infections, mortality, and testing rates. - Compare the US policy response to COVID-19 to other countries around the globe.	ILM Video lectures	HMS Course- Module 2 UCI COVID-19 Virology Video Lecture UCI Town Hall Video - “What you need to know.” Epidemiology Video- Exponential Growth Epidemiology Video- Simulating a Pandemic
**Radiology** The student will be able to describe indications for various COVID-19 imaging and recognize common imaging findings.	- List common radiograph findings of COVID-19 on chest radiograph (CXR). - List common CT chest findings of COVID-19. - Describe the sensitivity and specificity of CT and CXR in the diagnosis of COVID-19. - Synthesize a position statement of the use of CT imaging in diagnosis of COVID-19. - Describe the implications of radiology suite disinfection practices on resource utilization and patient care.	Video lectures Reading Evidence-based argument writing	Osmosis radiology video Reading international radiology position statements Radiology position statements
**Ventilator management** The student will be able to apply knowledge of COVID-19 pathophysiology to ventilator setting selection and troubleshooting.	- Describe the basic mechanics of a ventilator. - Interpret ventilator pressure and flow curves for common pathology. - Choose appropriate ventilator settings for lung injury. - Troubleshoot an alarming ventilator. - Describe the ARDSnet ventilation protocol. - Explain why ARDSnet ventilation protocol is the ideal ventilation strategy in COVID-19 patients. - Order the approach of escalating ventilator interventions for the decompensating COVID-19 patient. - Discuss emerging technologies to bridge resource needs in ventilator shortages.	Podcasts Team-based learning (TBL) Activity ILM	Emergency Medicine Reviews and Perspectives Podcast - Vents 101 Ventilator Management TBL HMS Course - Module 5
**Telehealth** The student will be able to demonstrate compassionate patient care through a tele-health delivery system.	- Define the term “telehealth.” - Describe the role of telehealth interventions in the COVID-19 crisis. - Compare telehealth patient care with traditional in-person patient care. - Demonstrate key telehealth interview skills.	Video lecture Simulation	Video: Panel Discussion from Telehealth Experts Standardized Patient Telehealth Small Groups
**Ethics, palliative care, and communication** Students will be able to analyze various ethical dilemmas related to the emergence of the COVID-19 pandemic.	- Identify various ethical dilemmas in patient care associated with the COVID-19 crisis. - Appraise the ethical dilemma of medical resource management during surge patient care during the COVID-19 crisis. - Prepare scripts for discussing end-of-life care of COVID-19 patients.	ILM Video Lecture Group Discussion Board	HMS Course - Module 4 Video Lecture: Ethics in a Pandemic Video Lecture: Interview with a Palliative Care Expert Discussion Board Activity: Initiating Palliative Care Discussions
**Evaluation of emerging literature** The student will be able to appraise and evaluate emerging COVID-19 literature.	- Appraise emerging literature related to the COVID-19 pandemic. - Collect evidence for/against the use of various PPE for healthcare professionals caring for COVID-19 patients. - Collect evidence for/against the use of hydroxychloroquine/azithromycin in COVID-19 treatment. - Justify the use/prohibition of NSAIDs in COVID-19 patients.	Debates	Discussion Board Debates: Controversies in COVID-19 Care.
**Patient and personal safety** The student will be able to demonstrate proper use of personal protective equipment.	- Differentiate between the various precautions (airborne, contact, droplet). - List the various respiratory PPE equipment and summarize the protections afforded by each piece of equipment. - Demonstrate the proper donning/doffing techniques for PPE used in COVID-19 patients. - Summarize the use of homemade PPE for healthcare providers.	Video lectures ILM Simulation	Video Lecture: Proper PPE Donning and Doffing at UCI Video Lecture: Osmosis N95 Video HMS Course - Module 5 COVID-19 simulated patient encounter
**Mental health and wellness** The student will be able to describe the stressors related to COVID-19 patient care and employ various mental health coping tools.	- List potential effect of quarantine during a pandemic on the mental health of patients. - Describe the various mental health tolls on providers caring for COVID-19 patients. - Employ various coping tools when working under the stressors of COVID-19 patient care.	Video lecture Group Discussion Board	HMS Course - Module 6 Video Lecture: Reflections from the Frontline - Panel Discussion with NYC Providers Discussion Board Activity: Mental Health in the Time of a Pandemic
**Service** The student will be able to show commitment to the Orange County, CA, community through COVID-19 related service efforts.	- Provide administrative, educational, and appropriate clinical support to the UCI health system in the face of the COVID-19 crisis.	Service Learning	Various community service activities arranged by students

*COVID-19*, coronavirus disease 2019; *SARS-CoV-2*, severe acute respiratory syndrome-coronavirus-2; *UCI*, University of California, Irvine; *CT*, computed tomography.

*ARDSnet*, acute respiratory distress syndrome network; *PPE*, personal protective equipment; *NSAID*, non-steroidal anti-inflammatory drug.

*COVID-19*, coronavirus disease 2019; *CA*, California.
