# Supplementary material for: An Effective COVID-19 Medical Student Elective
Source: West J Emerg Med. 2022 Jan 3;23(1):40–6. doi: 10.5811/westjem.2021.11.53656 (PMC8782135; doi:10.5811/westjem.2021.11.53656)
Supplement: Supplementary file 1 [file wjem-23-40-s001.docx]

Appendix 1: Course Objectives mapped to UCI School of Medicine program objectives and competencies.

The UCI School of Medicine organizes its program objectives into four core competencies denoted by one word (knowledgeable, skillful, altruistic and dutiful). Each core competency has multiple sub-competencies and specific program objectives. This table maps each objective from our course to the school of medicine program objectives.

| **Course objective** | **Core Competency** | **Sub Competency** | **Mapped UCI School of Medicine program objective(s)** |
| --- | --- | --- | --- |
| The student will be able to explain general and specific mechanisms by which the SARS-CoV-2 virus causes disease. | Knowledgeable | Disease Pathogenesis and Treatment | Knowledge of the pathogenesis of diseases, interventions for effective treatment, and mechanisms of health maintenance to prevent disease. |
| The student will be able to describe the epidemiology of the SARS-CoV-2 virus and explain how various public health interventions curb the spread of infection. | Knowledgeable | Population Health and Epidemiology | Knowledge of population health, epidemiology principles and the scientific basis of research methods relevant to healthcare. |
| The student will be able to recognize the clinical presentation of a COVID-19 patient, select and interpret diagnostic tests and explain interventions for effective treatment of COVID-19 patients. | Knowledgeable  Skillful | Disease Pathogenesis and Treatment  Patient Management | Knowledge of the pathogenesis of diseases, interventions for effective treatment, and mechanisms of health maintenance to prevent disease.  The ability to articulate a cogent, accurate assessment and plan, and problem list, using diagnostic clinical reasoning skills in all the major disciplines. |
| The student will be able to appraise and evaluate emerging COVID-19 literature. | Skillful  Dutiful | Evidence-Based Medicine  Lifelong Learning | The ability to search the medical literature, including electronic databases, and to locate and interpret up-to-date evidence to optimize patient care.  A commitment to lifelong learning and independently seeking new knowledge and skills in their own recognized areas of learning deficit. |
| The student will be able to analyze various ethical dilemmas related to the emergence of the COVID-19 pandemic. | Altruistic | Professionalism | Honesty and integrity reflecting the standards of the profession, in interacting with colleagues, patients, families and professional organizations. |
| The student will be able to describe the stressors related to COVID-19 patient care and employ various mental health coping tools. | Dutiful | Personal Well-being | A commitment to personal well-being and the well-being of family and friends. |
| The student will be able to show commitment to the orange county community through COVID-19 related service efforts. | Altruistic  Dutiful | Compassion  Cultural and Social Awareness  Community | Professional behaviors reflecting compassion and respect for patient privacy, altruism and a commitment to comprehensive, holistic medical care.  The commitment to seek knowledge and skills to better serve the needs of the underserved in their communities.  A commitment to serve our community. |
