# Supplementary material for: An Effective COVID-19 Medical Student Elective
Source: West J Emerg Med. 2022 Jan 3;23(1):40–6. doi: 10.5811/westjem.2021.11.53656 (PMC8782135; doi:10.5811/westjem.2021.11.53656)
Supplement: Supplementary file 2 [file wjem-23-40-s002.docx]

| \| Appendix 2- End of course evaluation \| \| --- \| \|  \| |
| --- | --- | --- |
| \|  \|  \| \| --- \| --- \| \|  \| After completing this course, please rate your satisfaction with the following:   \| \|  \| **Very Dissatisfied** \| **Dissatisfied** \| **Satisfied** \| **Very Satisfied** \| **N/A (Not Applicable)** \| \| --- \| --- \| --- \| --- \| --- \| --- \| \| Clarity of course educational objectives* \|  \|  \|  \|  \|  \| \| Overall growth of your clinical competence and knowledge* \|  \|  \|  \|  \|  \| \| Clarity and helpfulness of orientation* \|  \|  \|  \|  \|  \| \| Clarity and fairness of the evaluation/grading process* \|  \|  \|  \|  \|  \| \| Overall quality of instructional materials.* \|  \|  \|  \|  \|  \| \| Overall quality of faculty role modeling and clinical teaching* \|  \|  \|  \|  \|  \| \| Quality and quantity of ongoing, informal feedback* \|  \|  \|  \|  \|  \| \| \| --- \| --- \| --- \| --- \| --- \| --- \| --- \| --- \| --- \| --- \| --- \| --- \| --- \| --- \| --- \| --- \| --- \| --- \| --- \| --- \| --- \| --- \| --- \| --- \| --- \| --- \| --- \| --- \| --- \| --- \| --- \| --- \| --- \| --- \| --- \| --- \| --- \| --- \| --- \| --- \| --- \| --- \| --- \| --- \| --- \| --- \| --- \| --- \| --- \|  \| After completing this course, please rate your confidence in the following:   \|  \| **No Confidence** \| **Slight Confidence** \| **Moderate Confidence** \| **High Confidence** \| **N/A (Not Applicable)** \| \| --- \| --- \| --- \| --- \| --- \| --- \| \| Describing the global and local epidemiology of COVID-19. \|  \|  \|  \|  \|  \| \| Evaluating a patient for COVID-19 \|  \|  \|  \|  \|  \| \| Medically managing a patient diagnosed with COVID-19 \|  \|  \|  \|  \|  \| \| Managing ventilators in intubated patients \|  \|  \|  \|  \|  \| \| Donning and doffing personal protective equipment in a safe and effective manner \|  \|  \|  \|  \|  \| \| Communicating with patients over tele-health platforms \|  \|  \|  \|  \|  \| \| Discussing palliative care issues with patients \|  \|  \|  \|  \|  \| \| Critically evaluating emerging literature \|  \|  \|  \|  \|  \| \| Discussing and utilizing mental health resources related to stressors in providing care during a pandemic. \|  \|  \|  \|  \|  \|   Please rate your agreement with the following statements:   \|  \| **Strongly Disagree** \| **Disagree** \| **Agree** \| **Strongly Agree** \| **N/A (Not Applicable)** \| \| --- \| --- \| --- \| --- \| --- \| --- \| \| This course was valuable to my training as a future clinician. \|  \|  \|  \|  \|  \| \| This course was valuable to my training as a future clinician. \|  \|  \|  \|  \|  \| \| I plan to implement tools from this course into my future clinical practice. \|  \|  \|  \|  \|  \| \| \| --- \| --- \| --- \| --- \| --- \| --- \| --- \| --- \| --- \| --- \| --- \| --- \| --- \| --- \| --- \| --- \| --- \| --- \| --- \| --- \| --- \| --- \| --- \| --- \| --- \| --- \| --- \| --- \| --- \| --- \| --- \| --- \| --- \| --- \| --- \| --- \| --- \| --- \| --- \| --- \| --- \| --- \| --- \| --- \| --- \| --- \| --- \| --- \| --- \| --- \| --- \| --- \| --- \| --- \| --- \| --- \| --- \| --- \| --- \| --- \| --- \| --- \| --- \| --- \| --- \| --- \| --- \| --- \| --- \| --- \| --- \| --- \| --- \| --- \| --- \| --- \| --- \| --- \| --- \| --- \| --- \| --- \| --- \| --- \| --- \|  \|  \| **Strongly Disagree** \| **Disagree** \| **Agree** \| **Strongly Agree** \| \| --- \| --- \| --- \| --- \| --- \| \| Faculty, residents, students, and other members of the team treat each other professionally and with respect* \|  \|  \|  \|  \| \| Nurses and ancillary staff consistently treat students professionally and with respect \|  \|  \|  \|  \|  \| \|  \| **Very Dissatisfied** \| **Dissatisfied** \| **Satisfied** \| **Very Satisfied** \| **N/A (Not Applicable)** \| \| --- \| --- \| --- \| --- \| --- \| --- \| \| Organization, accessibility, and effectiveness of clerkship director \|  \|  \|  \|  \|  \| \| Organization, accessibility, and effectiveness of clerkship coordinator \|  \|  \|  \|  \|  \| \| \| --- \| --- \| --- \| --- \| --- \| --- \| --- \| --- \| --- \| --- \| --- \| --- \| --- \| --- \| --- \| --- \| --- \| --- \| --- \|  \| \| Did you experience any mistreatment or abuse from a faculty member, resident, fellow or staff member during your tenure in this course/clerkship? \| Yes \| No \| \| --- \| --- \| --- \| \| \| --- \| --- \| --- \| --- \|  \| \|  \| **I wish to report it below** \| **I do not wish to report at this time. Checking this item will provide non specific data for monitoring the course and will not trigger any followup response** \| \| --- \| --- \| --- \| \| If you have experienced or observed mistreatment (select one): \|  \|  \| \| \| --- \| --- \| --- \| --- \| --- \| --- \| --- \|  \| \| If so, please describe. \| \| --- \| \|  \| \| \| --- \| --- \| --- \|  \| \| GENERAL NARRATIVE COMMENTS: (Please be professional, constructive and specific). \| \| --- \| \|  \| \| \| --- \| --- \| --- \| \| \|  \|  \| \|  \|  \| \|  \|  \| |
