# Supplementary material for: An Effective COVID-19 Medical Student Elective
Source: West J Emerg Med. 2022 Jan 3;23(1):40–6. doi: 10.5811/westjem.2021.11.53656 (PMC8782135; doi:10.5811/westjem.2021.11.53656)
Supplement: Supplementary file 3 [file wjem-23-40-s003.docx]

Appendix 3: Sample assignments and grading rubrics

**Discussion Board Assignments:**

Active participation is a must in this course. Various discussion questions, activities, debates, etc. will be posted. Generally, you will be required to respond to the main discussion question and then also make comments on two of the responses of others in the course. Please note that making the minimum number of postings should not be your only goal. The quality of your postings is critically important. Furthermore, you are expected to participate throughout the day in the discussions, not all at one time.

It is expected that you will fully participate in the online discussions. This means posting your own thoughts about the weekly topics, commenting on others’ ideas, and responding to questions about your own postings. Class participation points will be based on whether or not you contribute meaningfully and adequately to the class conversations. Both the quality and the quantity of your postings will be considered. While it is relatively easy to post numerous, non-substantive comments, it takes more thought and effort to post intelligent, meaningful comments that move the discussion forward. For example, meaningful posts tend to:

• Provide concrete examples, perhaps from your own experience
• Identify consequences or implications
• Challenge something that has been posted – perhaps by playing devil’s advocate
• Pose a related question or issue
• Suggest a different perspective or interpretation
• Pull in related information from other sources and cite the sources– books, articles, websites, courses, etc.

Consider your time commitment to our online discussions to be critical to your success as a learner, as well as to the success of the course. Participation scores will be based on three primary criteria:
1. Frequency and timeliness of postings
2. Content of your postings (the thoughtfulness/reflection that goes into your responses and the extent to which they address the topic, including the assigned readings)

Mental Health Discussion Topic:

After the required reading/podcast and watching "Reflections from NY" panel, please answer the following prompt:

Identify specific stressor (either mentioned in readings/video or not mentioned yet) that has resonated with you as you potentially care for COVID-19 patients in the future. Can you share any tips, apps, or resources to fellow classmates on stress management during this time?

Palliative Care Discussion Topic:

You are an outpatient geriatric provider at UCI Medical Center. Ms. Garcia is a 77 year old woman with history of morbid obesity, DM, HTN, CAD status post CABG 2 years prior who is presenting for difficult to control blood sugars. After changing her insulin regimen, you decide to approach a conversation about advanced directives and goals of care. She is accompanied by her son who lives with her and is her primary caretaker. As you start the conversation, he abruptly yells, "Why are you bringing this up now! She's fine! She doesn't even have a cough or fever!!"

How will you proceed with this conversation? Use examples of language you would use to de-escalate tensions with Ms. Garcia's son? How would you explain that this time and place is an appropriate place to have this conversation?

Community Service Discussion Topic:

A requirement of this two week elective is that students will participate in community service activities directly related to COVID-19's effects on the community and healthcare system.

In this discussion board, please answer the following questions regarding your community service participation:

1) Please describe your community service activity.

2) What need did your activity serve?

3) What is one thing that surprised you about your service activity?

4) How did your activity deal with physical distancing?

5) For a student taking this course after you, what next steps would you recommend to a volunteer to make the experience more effective for those you are serving?

6) Why do you think service is important during a time of crisis?

Discussion Rubric:


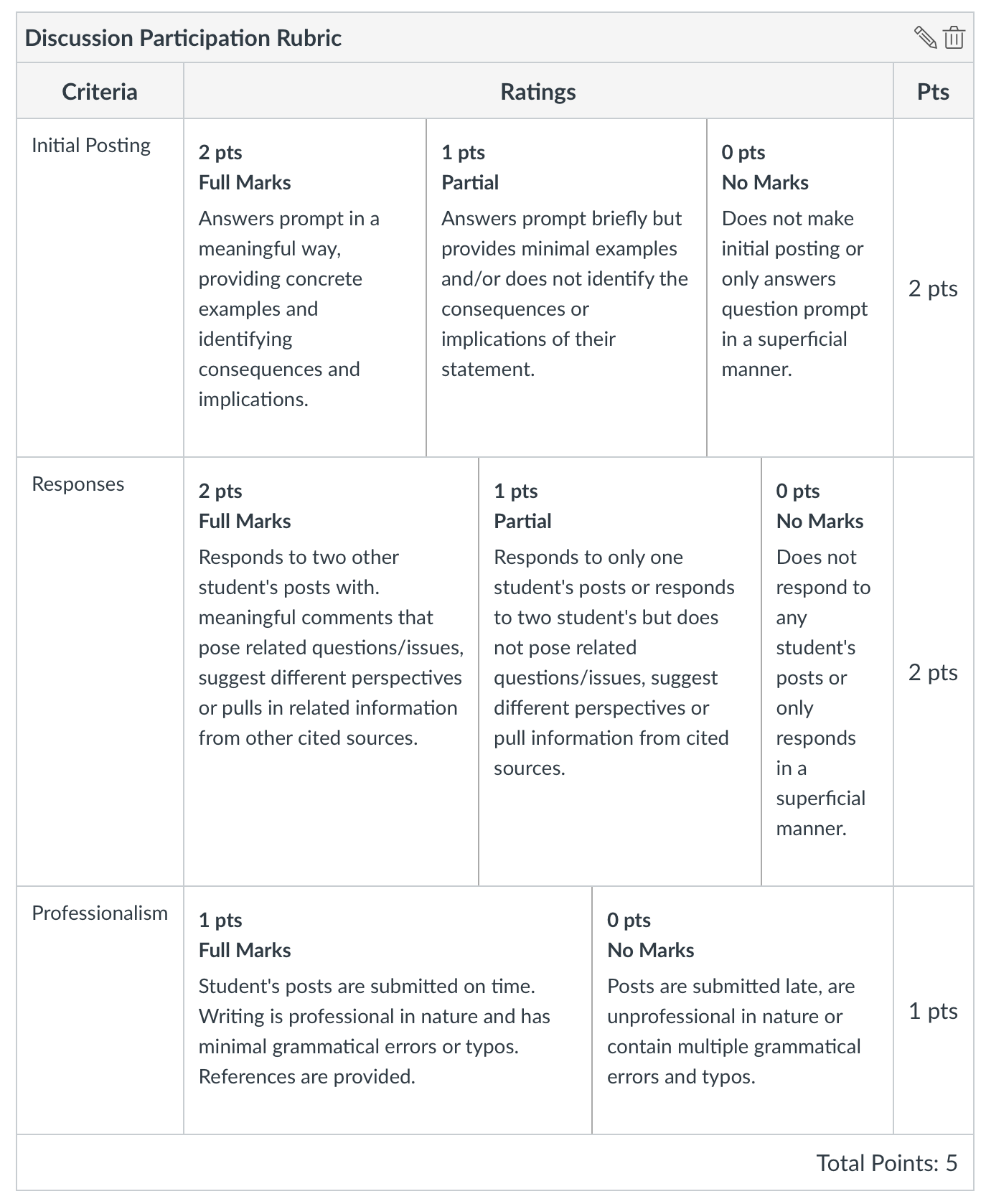


**Position Statement Assignment:**

The OC Board of Education recently voted 4-1 that schools should re-open in the fall without masking or social distancing in the classroom. You have been called upon as the Orange County Medical Director to respond to this vote.

In 500 words or less, please provide a position statement on school reopening. Your statement should reference the latest literature and address the following questions:

- What is the risk of infection on children and the risk of transmission to parents?
- Should schools re-open in the fall based on our current epidemiological data?
- If re-opening schools in inevitable, what modifications would you recommend to take place in the classroom?
- How can teachers remain protected during this time?

Please note your work **must**include outside evidence/literature not provided from this course. Please provide inline references in your work.

Rubric:


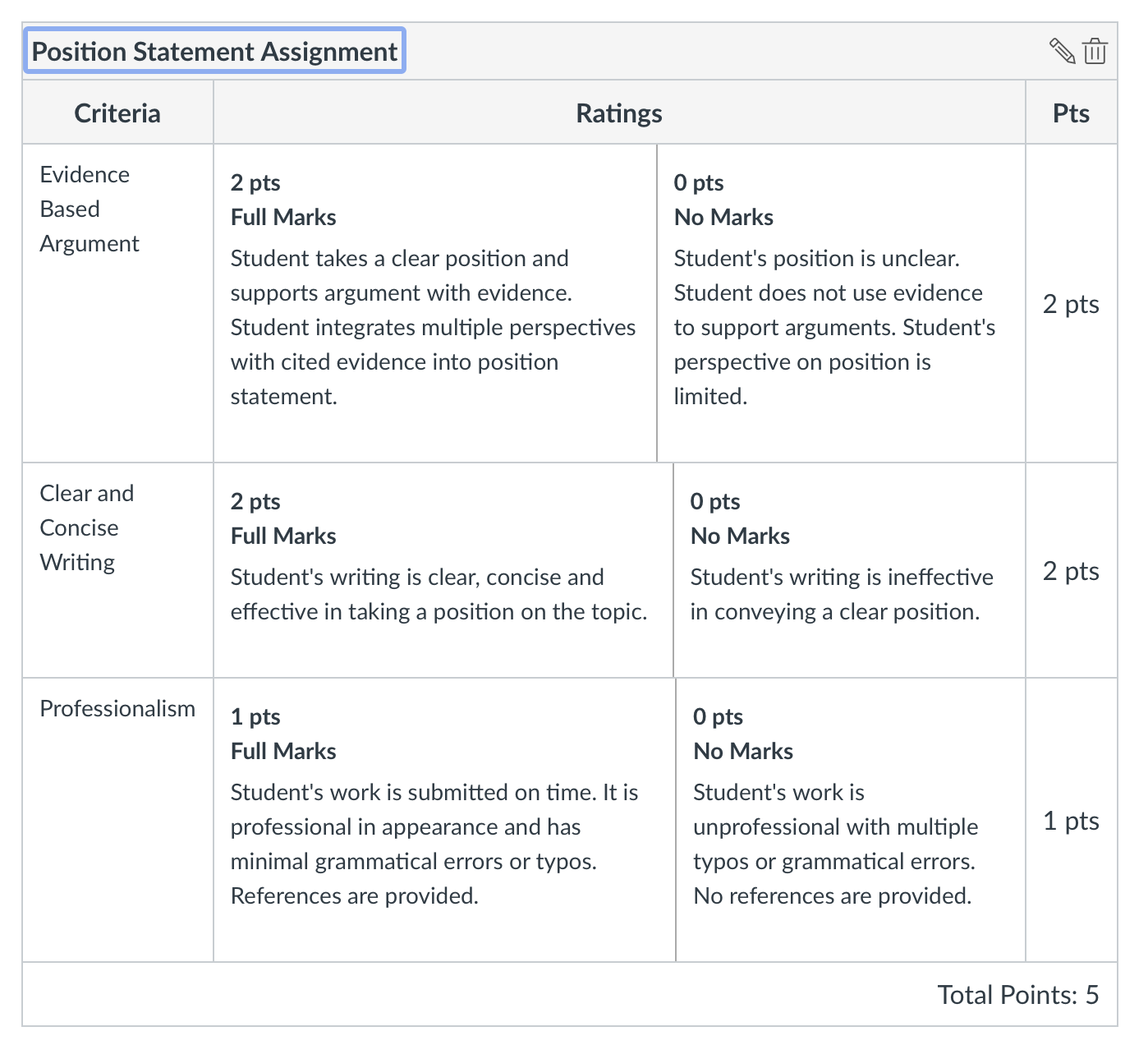


**Emergency Literature Debate Assignment**

Clinical care during a global pandemic requires rapid evaluation of emerging literature to make rational, evidence based decisions in care. For this exercise, you will be assigned a controversial topic related to COVID-19 care. Within that topic, you will either be assigned to defend one "side" of that controversial issue. You will perform a primary literature search to find evidence supporting your side of the argument.

For this assignment, please submit a **one-page position summary or info-graphic**supporting your argument. Please provide primary references indoor summary, citing evidence. The goal of this summary is to convince the audience of an argument grounded in evidence.

Debate Assignments:

- N95 vs Simple Facemask for healthcare workers
- Early vs Delayed intubation in hypoxic COVID-19 patients
- Hydroxychloroquine as an effective treatment for COVID-19
- Avoidance of ibuprofen in COVID-19 positive patients

Rubric:


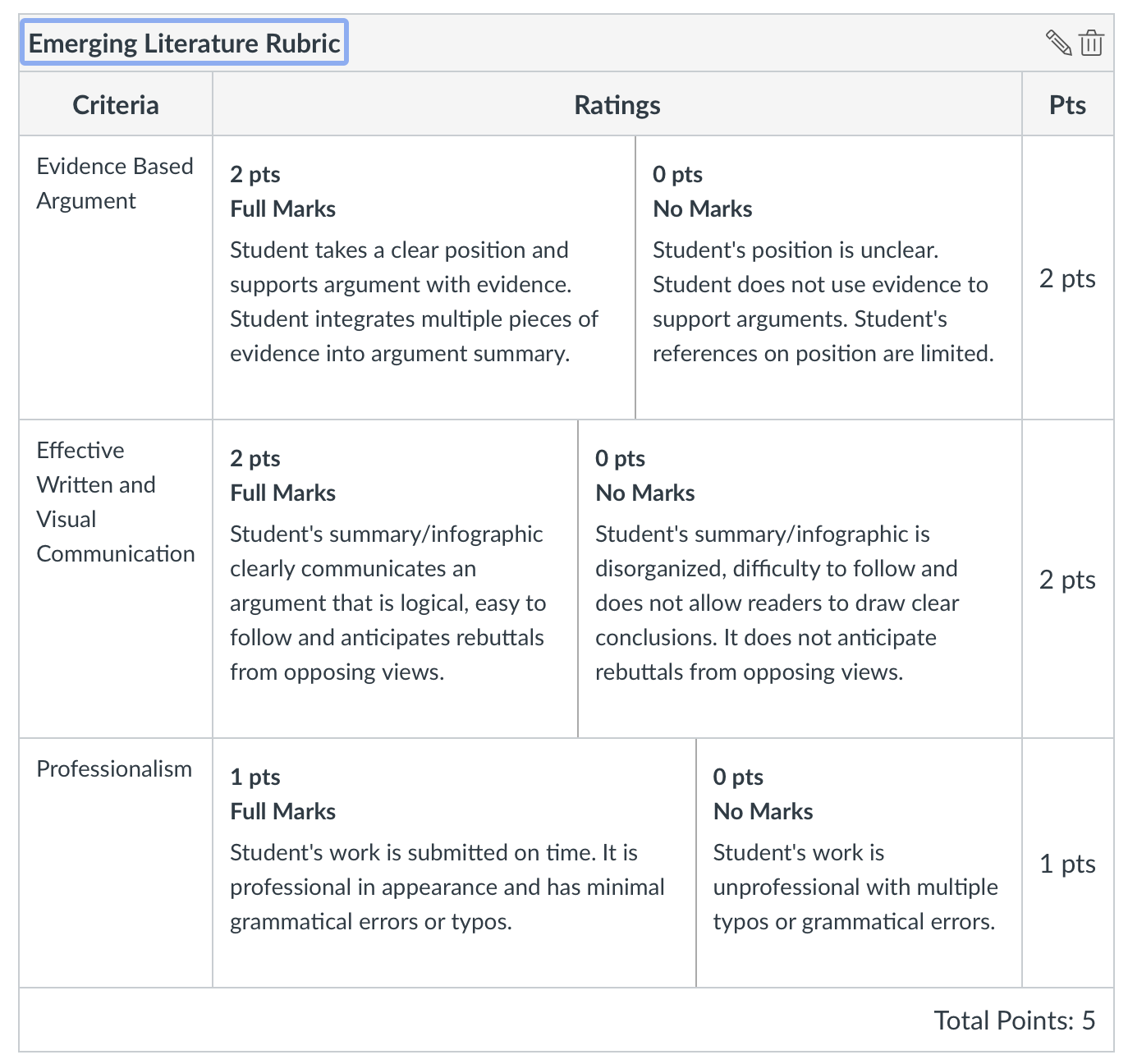


**Data Visualization Assignment:**

Using publicly available data, you will create a novel visualization/graphic portraying relevant data on COVID-19 cases in Orange County. The visualization can show any data including case growth, mortality rates, geographical distribution, testing numbers, populations affected, etc.

You can also consider comparing OC to other counties or similar populations globally.  Maps, graphs, video or other relevant media can be used. We know you are not graphic designers, but just want you to practice communicating interesting data. Excel, PowerPoint, Google Suite, Adobe Suite, Piktochart and Canva are all excellent programs to develop your visualization.

Examples of visualizations can be seen here:

[https://www.reddit.com/r/COVID19_data/ (Links to an external site.)](https://www.reddit.com/r/COVID19_data/)

[https://www.latimes.com/projects/california-coronavirus-cases-tracking-outbreak/ (Links to an external site.)](https://www.latimes.com/projects/california-coronavirus-cases-tracking-outbreak/)

Please note these are all outstanding examples used to inspire your work. They do not necessarily need to be this complex or in depth. They simply need to convey interesting information.

Rubric:


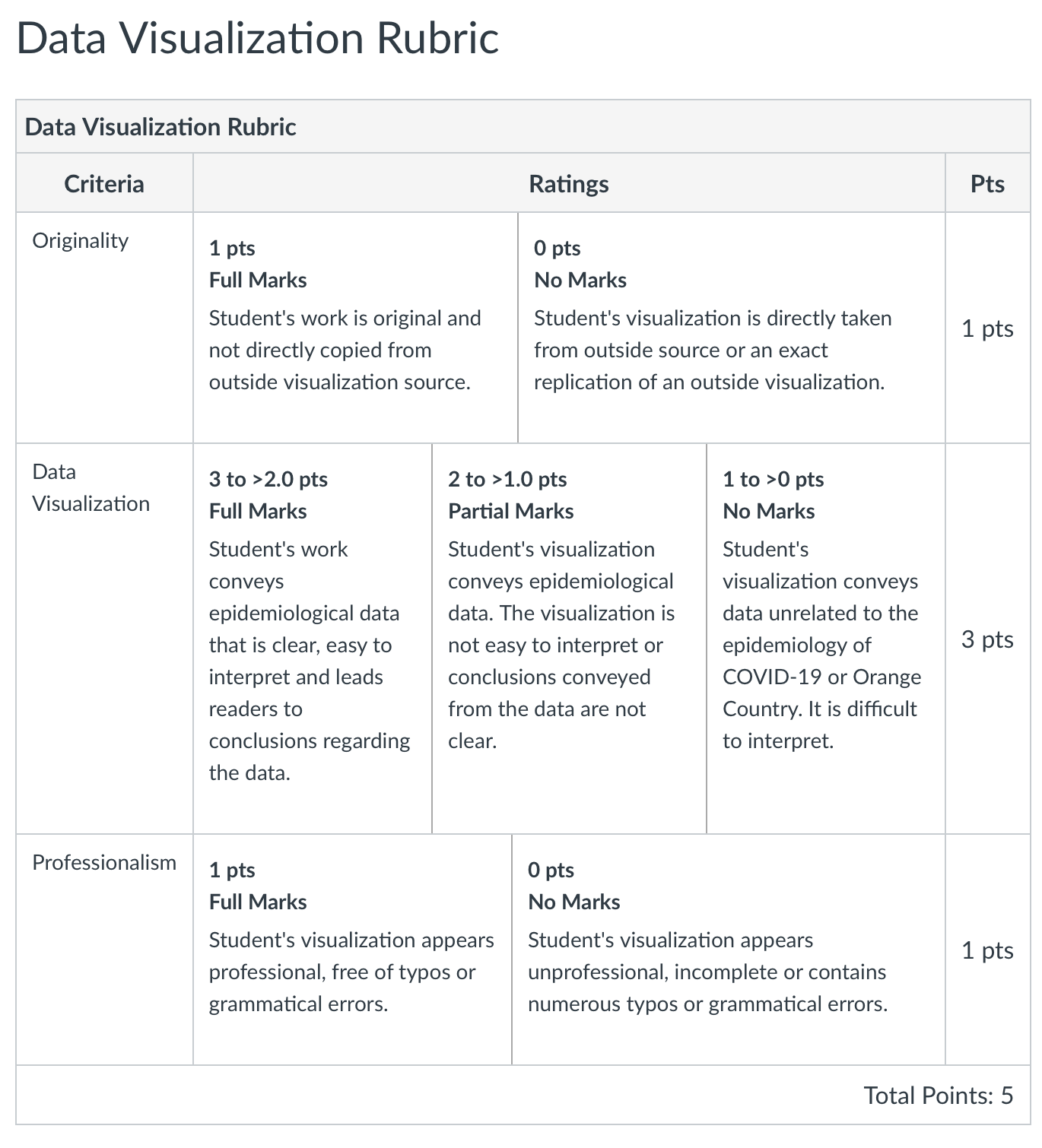


**Standardized Patient Encounters:**

Below are the critical actions checklist for each of the two tele-health, standardized patient encounters that students participated it.

Case 1:

| Action | Y | N | Comments |
| --- | --- | --- | --- |
| Student takes thorough history including history of present illness, past medical history, medications, allergies and social history including employment and living situation. |  |  |  |
| Student asks screening questions for possible COVID-19 symptoms including: cough, shortness of breath, fevers, myalgias, nausea, vomiting and diarrhea. |  |  |  |
| Student asks screening questions about possible exposure to COVID-19 including travel history, sick contacts or visitors from high risk areas. |  |  |  |
| Student is able to address and name the main concerns of Jason, reflecting their deep concern/anxiety over COVID-19. |  |  |  |
| Student reassures SP that symptoms are less concerning for COVID-19. |  |  |  |
| Student clearly explains why patient is not eligible for a test at this time. |  |  |  |
| Student is able to provide answers for next steps at home for the patient, including guidance on returning to work and quarantine. |  |  |  |
| Student is still able to provide good eye contact and demonstrate excellent communication skills through a video platform. |  |  |  |

Case 2:

| Action | Y | N | Comments |
| --- | --- | --- | --- |
| Student inquires on patient’s current clinical status including symptoms of fever, shortness of breath, cough and myalgias. |  |  |  |
| Student recaps what he/she knows about patient’s situation. |  |  |  |
| Student delivers positive test disclosure in an empathetic manner. |  |  |  |
| Student inquiries about patient’s fears/concerns with this positive diagnosis and addresses them. |  |  |  |
| Student reviews return precautions for red flag symptoms that patient would need to return to the health care setting. |  |  |  |
| Student empathizes with patient’s fear regarding positive diagnosis in the setting of critically ill brother. |  |  |  |
| Student provides clear guidance on patient’s need to quarantine away from family/loved ones. |  |  |  |
| Student inquires if there are any resources/needs that could be provided to the patient. |  |  |  |
